# The time course of calf muscle fluid volume during prolonged running

**DOI:** 10.14814/phy2.14414

**Published:** 2020-05-06

**Authors:** Steffen Willwacher, David A. Sleboda, Daniela Mählich, Gert‐Peter Brüggemann, Thomas J. Roberts, Grischa Bratke

**Affiliations:** ^1^ Institute of Biomechanics and Orthopaedics German Sport University Cologne Cologne Germany; ^2^ School of Human Movement and Nutrition Sciences The University of Queensland St Lucia Queensland Australia; ^3^ Department of Ecology and Evolutionary Biology Brown University Providence RI USA; ^4^ Department of Diagnostic and Interventional Radiology University of Cologne Cologne Germany

**Keywords:** fluid volume, muscle, passive tension, running mechanics, triceps surae

## Abstract

Muscle fluid is essential for the biochemistry and the biomechanics of muscle contraction. Here, we provide evidence that muscle fluid volumes undergo significant changes during 75 min of moderate intensity (2.7 ± 0.4 m/s) running. Using MRI measurements at baseline and after 2.5, 5, 10, 15, 45 and 75 min, we found that the volumes of calf muscles (quantified through average cross‐sectional area) in 18 young recreational runners increase (up to 9% in the gastrocnemii) at the beginning and decrease (below baseline levels) at later stages of running. However, the intensity of changes varied between analyzed muscles. We speculate that these changes are induced by muscle activity and dehydration‐related changes in osmotic pressure gradients between intramuscular and extramuscular spaces. These findings highlight the complex nature of muscle fluid shifts during prolonged running exercise.

## Graphical abstract

We provide evidence that muscle fluid volumes undergo significant changes during 75 min of moderate‐intensity (2.7 ± 0.4 m/s) running. Using MRI measurements, we found that the volumes of calf muscles (quantified through average cross‐sectional area) in 18 young recreational runners increase (up to 9% in the gastrocnemii) at the beginning and decrease (below baseline levels) at later stages of running. These findings highlight the complex nature of muscle fluid shifts during prolonged running exercise.

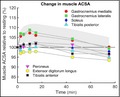

## 1 INTRODUCTION

Water is an essential component of the human body with important metabolic, transport, temperature control, structural, and mechanical functions (Lorenzo, Serra‐Prat, & Yébenes, 2019). In skeletal muscle, for example, fluid flows are crucial in the regulation of ion concentrations and pH, which affect muscle contraction and hence force development during exercise (Sjogaard & Saltin, 1982). In addition to the importance of muscle fluids for the biochemistry of muscle contraction, recent studies suggest that fluid plays important mechanical roles within muscle, influencing passive force development during stretch (Gindre, Takaza, Moerman, & Simms, 2013; Sleboda & Roberts, 2017), work production during active contraction (Azizi, Deslauriers, Holt, & Eaton, 2017), and dynamic changes in muscle shape that influence muscle speed and force (Azizi, Brainerd, & Roberts, 2008; Eng, Azizi, & Roberts, 2018).

It is well documented that with the onset of muscle activity, fluid moves from blood plasma into muscle due to increased hydrostatic (Kjellmer, 1964) and osmotic (Lundvall, Mellander, Westling, & White, 1972) forces. These findings have been confirmed in sympathectomized cat muscle (Björnberg, 1990), in perfused, isolated cat muscle (Ward, Hamilton, & Watson, 1996; Watson, Garner, & Ward, 1993) and frog muscle (Damon et al., 2002). Furthermore, when stimulating single muscle fibers, an initial rapid increase in A‐Band spacing, indicating an increase in muscle cell volume is followed by a slower convergence towards a plateau with continued stimulation (Rapp, Ashley, Bagni, Griffiths, & Cecchi, 1998).

Activity‐induced increases in muscle volumes have also been observed in vivo in different kinds of (sport) activities, for example, after short‐term, high‐intensity exercises (Ploutz‐Snyder, Convertino, & Dudley, 1995; Raja, Raymer, Moran, Marsh, & Thompson, 2006; Shi, Zheng, Chen, & Huang, 2007; Sjogaard & Saltin, 1982). Furthermore, increased muscle thickness was observed after a prolonged isometric contraction of the supraspinatus muscle (Jensen, Jørgensen, & Sjøgaard, 1994).

Additional evidence for the shift of fluids into the intracellular space results from the proton transverse relaxation time (T_2_) increases observed by magnetic resonance imaging after exercise (Meyer & Prior, 2000). Increases in T_2_ times with exercise have been frequently reported in the literature (Fisher, Meyer, Adams, Foley, & Potchen, 1990; Fleckenstein et al.,; Shellock, Fukunaga, Mink, & Edgerton, 1991). It has been suggested that increases in T_2_ relaxation times are related to fluid content within a tissue, even though the exact mechanism underlying T_2_ time increases with muscle activity is not well understood (Archer et al., 1992; Cole, Leblanc, & Jhingran, 1993; Fleckenstein et al., 1991; Ploutz‐Snyder, Nyren, Cooper, Potchen, & Meyer, 1997). However, T_2_ changes cannot quantify absolute muscle volume increases due to fluid shifts. Consequently, segmentation of muscle outlines within MRI images is currently considered the gold standard for muscle volume determination (Pons et al., 2018). Furthermore, this method has been proven to be able to detect intervention‐related changes in leg muscle volumes (Hudelmaier et al., 2010).

During prolonged running, it was found that total muscle water of the vastus lateralis muscle increased at 10 min and then stayed almost constant at 120 min (Costill, Coté, Fink, & Handel, 1981). However, neither this nor other studies addressing fluid shifts during prolonged running exercise (Senay & Pivarnik, 1985) have analyzed the time course of muscle fluid volume changes at a higher temporal resolution. Furthermore, despite the importance of calf muscles for propulsion and support during running (Hamner, Seth, & Delp, 2010), analyses of volume changes in these muscles during locomotion are rare. This gap of knowledge limits our current understanding of how muscle fluid volumes of calf muscles change in distance running.

Therefore, the purpose of the present study was to describe the time course of muscle volume changes during a prolonged bout of running. Based on results from single fiber stimulation (Rapp et al., 1998) and results from T_2_‐relaxation time changes after exercise, we hypothesized that whole‐organ muscle volumes would increase rapidly with the onset of activity and then approach a plateau at a slower rate.

## 2 MATERIALS AND METHODS

We included 18 recreationally active participants (11 men and 7 women; height: 1.77 ± 0.08 m; body mass: 70.6 ± 7.5 kg; age: 29 ± 3 years) in this study. All participants were free of injuries for at least 12 months before the study. The study protocol was approved by the local university ethics committee. All procedures were carried out in compliance with the Declaration of Helsinki. Before the data collection, we obtained written, informed consent from all participants.

**FIGURE 1:**
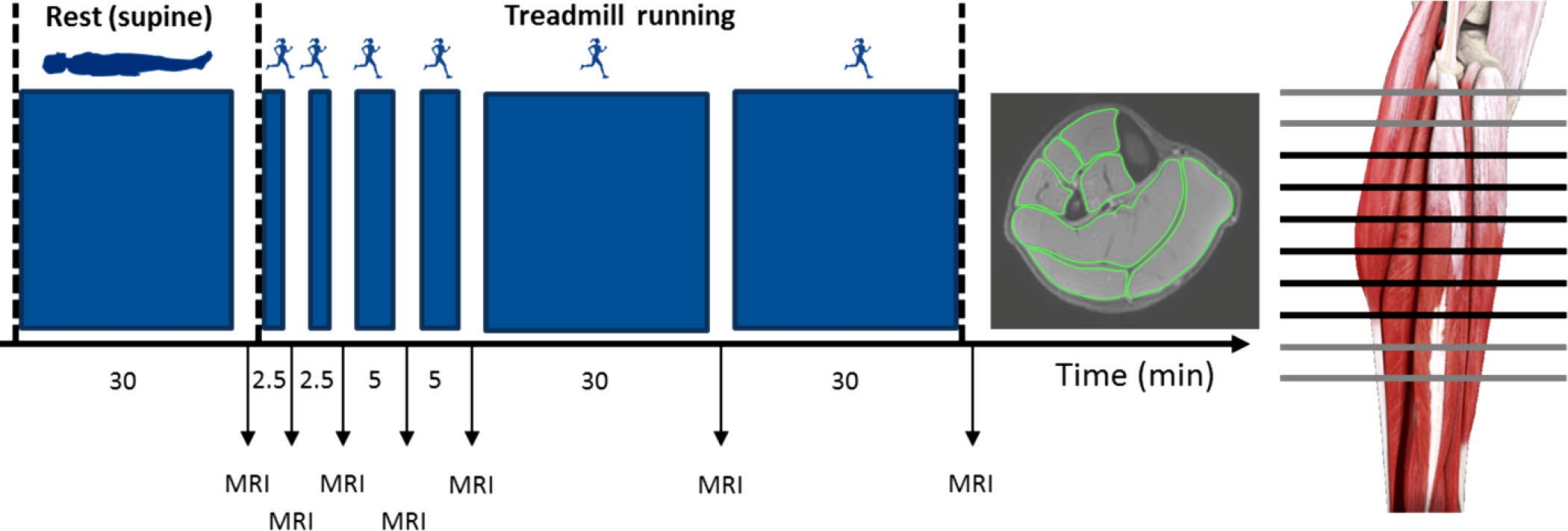
Protocol of the study. Black arrows mark the time points of magnetic resonance imaging (MRI) measurements. Vertical black broken lines indicate (from left to right) the start of the protocol, the onset of running and the end of running. The right part of the image highlights the location of slides taken using the 3.0‐Tesla MRI machine. Note: we only analyzed the black slides for the purposes of this study. In the middle part of the Figure, we provide an example of the digitized outlines of the muscles

All participants underwent a protocol consisting of 30 min of resting in a supine position to achieve an uninfluenced baseline volume followed by in total 75 min of running at a self‐selected speed on a treadmill (Figure 1). The running speeds were selected individually such that they could be kept for the entire 75‐min running protocol. Running speeds varied between 2.2 and 3.6 m/s (mean: 2.7 m/s; standard deviation: 0.4 m/s) between participants. The treadmill was positioned directly in front of the MR room (about 10 m) with a 3.0‐Tesla magnetic resonance imaging (MRI) scanner (Philips Ingenia 3.0T, Philips Healthcare, Best, Netherlands). A 16‐channel knee coil was chosen to achieve the best possible resolution as well as fixation and reproducibility of the positioning. In addition, the feet were fixed to a plate connected to the MR Table to allow fast and accurate repositioning between scans. A T2 spin echo sequence was used for the anatomical delineation of the different muscles (echo time: 10 ms; repetition time: 2000 ms; flip angle 90 degree; field of view: 150 × 197 × 185 mm; slice thickness: 5 mm; number of slices: 10; gap: 15 mm; acquisition voxel: 1.5 × 1.5 × 1.5 mm; reconstruction voxel: 0.61 × 0.61 mm; turbo factor: 16; parallel imaging acceleration factor (SENSE): 1.7). In previous studies, both T1‐ and T2‐weighted sequences have been successfully validated for muscle segmentation (McColl, Fleckenstein, Bowers, Theriault, & Peshock, 1992; Orgiu et al., 2016; Warfield, Mulkern, Winalski, Jolesz, & Kikinis, 2000). T2‐weighted sequences allow not only the anatomical visualization of the muscles but also a subjective assessment of a possible delimitable intramuscular edema as a potential correlate for volume increase. Two saturation slabs (cranial and caudal the field of view) were used to decrease artifacts due to inflowing blood. The cranio‐caudal field of view was 15 cm, starting proximally at the fibular head. The scanning time was 2:16 ± 0:27 min, additionally it took 0:42 ± 0:09 min between pause of the run and start of the scan as well as further 0:42 ± 0:18 min after completion of the scan until the restart of the run. We performed MRI scans of the shank before and after 2.5, 5, 10, 15, 45, and 75 min of running in a standardized ankle and knee joint configuration (Figure 1). The run was only interrupted for the duration of the scans and resumed with the same speed. The ankle joint was positioned neutrally (90° joint angle) while the knee was positioned in a slightly (about 20°) flexed configuration. In this position, we took ten 5‐mm thick transversal MRI slice images of the shank.

To quantify muscle volume, a board‐certified radiologist with special focus on musculoskeletal imaging (6 years of experience) manually segmented the medial (GM) and lateral gastrocnemius (GL), soleus (SO), peroneus longus (PER), tibialis posterior (TP), tibialis anterior (TA), and extensor digitorum longus (EDL) muscles within each MRI slice and for every time point. Similar to Ploutz‐Snyder et al. (Ploutz‐Snyder et al., 1995), we quantified muscle volumes through the average cross‐sectional areas of each muscle. For this step, we excluded the two most proximal and two most distal slices, since they were contaminated with artifacts in some participants and muscles.

We performed one factor (time) repeated measures analysis of variance (ANOVA) in order to identify the effects of running time on the muscles anatomical cross‐sectional area (ACSA). In case of a significant main effect of time, we performed post hoc comparisons between time points while using Tukey's Honestly Significant Difference procedure to control for alpha‐error accumulation.

## 3 RESULTS

**FIGURE 2:**
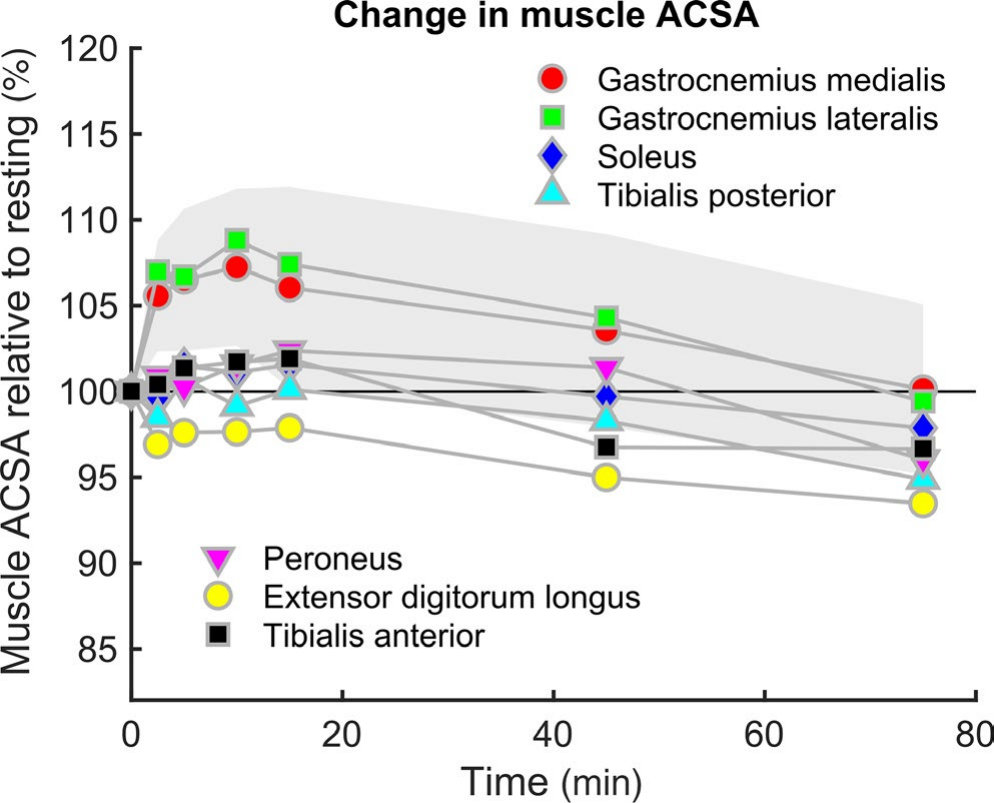
Time course of relative changes (baseline = 100%) in average muscle cross‐sectional area as a function of running time. Each data point represents the mean of all runners for the respective muscle. The gray area indicates ± one standard deviation around the mean of the gastrocnemius medialis condition

We observed a significant increase in ACSA of more than 5% compared to the baseline of both gastrocnemius muscles after 2.5 min of running (*p* < .032; Figure 2; Table 1). Subsequently, ACSAs of the gastrocnemii further increased at a slower rate and reached peak values of about 109% and about 107% of resting values after 10 min of running for gastrocnemius lateralis and gastrocnemius medialis, respectively (Figure 2; Table 1). We observed a similar pattern of the initial increase in soleus, tibialis anterior, and peroneus longus muscles. However, the amplitudes of ACSA changes were much smaller in these muscles (< 3%), and we found no significant differences compared to baseline (Figure 2; Table 1). Tibialis posterior and extensor digitorum longus showed no initial increase in ACSA (Figure 2; Table 1).

**TABLE 1:** Average (± one *SD*) cross‐sectional areas of the analyzed muscles at the respective time points

Time (min)	Gastrocnemius lateralis (cm^2^)	Gastrocnemius medialis (cm^2^)	Soleus (cm^2^)	Peroneus longus (cm^2^)	Tibialis posterior (cm^2^)	Tibialis anterior (cm^2^)	Extensor digitorum longus (cm^2^)
0	5.3 ± 1.7^2.5,5,10,15^	10.6 ± 2.6^2.5,5,10,15^	16.2 ± 3.3	4.6 ± 1.1	2.8 ± 0.7	4.7 ± 0.8	2.4 ± 0.6
2.5	5.7 ± 2.0^0,75^	11.2 ± 2.9^0,75^	16.1 ± 3.4	4.6 ± 1.2	2.7 ± 0.7	4.7 ± 0.8	2.3 ± 0.6
5	5.7 ± 2.0^0,75^	11.3 ± 2.8^0,75^	16.4 ± 3.5	4.6 ± 1.2	2.8 ± 0.7^75^	4.8 ± 0.9	2.4 ± 0.6
10	5.8 ± 2.0^0,75^	11.4 ± 2.9^0,75^	16.3 ± 3.4	4.6 ± 1.2^75^	2.7 ± 0.8	4.8 ± 0.9^75^	2.4 ± 0.6
15	5.7 ± 2.0^0,75^	11.3 ± 2.9^0,75^	16.4 ± 3.5	4.7 ± 1.1^75^	2.7 ± 0.7^75^	4.8 ± 0.9^75^	2.4 ± 0.6
45	5.7 ± 1.8^0,75^	11.0 ± 2.8	16.1 ± 3.4	4.6 ± 1.1	2.7 ± 0.7	4.6 ± 0.9	2.3 ± 0.6
75	5.3 ± 1.8^2.5,5,10,15,45^	10.6 ± 2.7^2.5,5,10,15^	15.9 ± 3.5	4.4 ± 1.1^10,15^	2.6 ± 0.7^5,15^	4.5 ± 0.8^10,15^	2.3 ± 0.6

Note

^0, 2.5, 5, 10, 15, 45^ and ^75^ indicate a significant (*p* < .05) difference to the volume at baseline, 2.5 min, 5 min, 15 min, 45 min and 75 min of running, respectively.

After having reached peak ACSA values between 5 and 15 min of running, all muscles showed a reduction over the later stages of the run (Figure 2, Table 1). For all muscles except gastrocnemius medialis, ACSAs were lower compared to baseline after 75 min of running. Except for soleus and extensor digitorum, the ACSAs after 75 min of running were significantly (*p* < .05) lower compared to the maximum ACSA observed within the first 15 min of running (Figure 2; Table 1).

## 4 DISCUSSION

The purpose of the present study was to provide a detailed insight into the time course of calf muscle volumes during prolonged running. We found increases in gastrocnemii ACSA of up to 9% above resting baseline in the early stages of the 75‐min run and reductions of up to 4% below baseline at the end of the running protocol(for the tibialis posterior muscle). Therefore, we can only partly accept that muscle volumes would increase rapidly with the onset of activation and then approach a plateau at a slower rate, since we observed ACSA reductions instead of a plateau in the later stages of the running protocol.

The fluid exchange between extramuscular and intramuscular compartments is a complex interplay between hydrostatic and osmotic forces, governed by anatomical and biochemical factors (Lorenzo et al., 2019; Senay & Pivarnik, 1985; Sjogaard & Saltin, 1982). In line with previous literature, we found a rapid initial increase of ACSA with the onset of exercise after 2.5 min of running. Several studies have reported that with the onset of muscle activity, the hydrostatic pressure gradient from the vascular towards the interstitial space of muscle rapidly drops, which theoretically would cause an efflux of fluid from muscle towards the vascular space (Folkow, Haglund, Jodal, & Lundgren, 1971; Höjensgàrd & Stürup, 1953; Nicolaides & Zukowski, 1986; Pollack & Wood, 1949). However, these changes in hydrostatic pressures are offset by alterations in osmotic pressure gradients as a result of muscular activity and the accumulation of exercise‐related metabolites (Björnberg, 1990; Lundvall et al., 1972; Meyer & Prior, 2000). The intensity of this opposing osmotic pressure gradient is considered to be proportional to the intensity of muscle action (Jenner, Foley, Cooper, Potchen, & Meyer, 1994). Senay and Pivarnik (Senay & Pivarnik, 1985) conclude from their own (Senay, 1970, 1972) and the work of others (Beaumont, Underkofler, & Beaumont, 1981; Greenleaf et al., 1977) that the osmotic pressure gradient exceeds the opposing hydrostatic pressure gradient approximately when “muscle exercises at 40–60 percent of its maximum oxygen consumption". We did not obtain the maximum rate of oxygen uptake (${\dot{V}O}_{2}\max$) of the runners in our study. However, when using the regression equation developed from a very similar sample of recreational runners to predict the oxygen uptake from treadmill running speed, and by considering the average ${\dot{V}O}_{2}\max$ of these runners (Williams & Nute, 1983), we can estimate that our runners were running at approximately 58% of their ${\dot{V}O}_{2}\max$. Consequently, fluids might have shifted from extracellular compartments (mostly from blood plasma (Ploutz‐Snyder et al., 1995; Senay & Pivarnik, 1985), towards interstitial and intracellular spaces due to increased osmotic drive towards the muscle.

We observed differences in the initial effects of running on ACSA between muscles, where both gastrocnemius muscles pronouncedly increased their ACSA, while peroneus, tibialis anterior, and soleus increased their ACSA to a lesser extent. Tibialis posterior and flexor digitorum longus did not increase or even decreased their ACSA (Figure 2). These differences could result from multiple factors. Since the increase in ACSA is related to the relative work rate of a muscle (Adams, Duvoisin, & Dudley, 1992; Fisher et al., 1990; Jenner et al., 1994), different relative involvement of muscles might explain these differences in part. Furthermore, the accumulation of metabolites within muscle cells is related to fiber type distribution (Prior, Ploutz‐Snyder, Cooper, & Meyer, 2001). Since gastrocnemius muscles contain a higher relative volume of fast‐twitch muscle fibers as compared to, for example, the soleus muscle (Alway, MacDougall, Sale, Sutton, & McComas, 1988), it is likely that fiber type distribution is one of the underlying factors explaining the more pronounced shift of fluid to the gastrocnemii observed in the initial stages of running (Stallknecht, Vissing, & Galbo, 1998).

Furthermore, the anatomical arrangement of muscles within the leg may influence fluid movement. The gastrocnemius, for example, is arranged superficial to the deeper soleus and tibialis posterior muscles. Muscles exert transverse forces on each other during contraction (Siebert, Günther, & Blickhan, 2012), and we speculate that deeper muscles may experience higher intramuscular pressures that might oppose fluid influx. These factors remain speculative, however, and will require experimental verification through future studies.

Beyond 15 min of running, the ACSA of all analyzed muscles began to decrease, and by the end of the running trials, all muscles but the soleus had lower ACSA values compared to baseline measurements. We believe that sweat losses of total body water might be an important factor explaining some of the almost linear reduction in muscle ACSA. Sweat losses can affect the osmolality of blood (Duvillard, Braun, Markofski, Beneke, & Leithäuser, 2004). Hence, with accumulated sweat losses, the osmotic pressure gradient might change with prolonged running such that the overall pressure gradient (combined osmotic and hydrostatic) favors movement of water from muscle to the vascular space. With prolonged running, fluids likely shift from intracellular and interstitial muscle spaces to the vascular space in order to conserve blood plasma volume and to protect vital function (Costill, 1977; Costill et al., 1981; Nose, Mack, Shi, & Nadel, 1988).

Next to sweating‐related changes in osmotic pressure gradients, it is conceivable that changes in metabolite contents within intra‐ and extramuscular spaces induce osmotically driven fluid shifts away from active muscle tissues. With prolonged submaximal exercise, it has been observed that lactate is removed from active muscle to the vascular space at a higher rate, potentially leading to changes in osmotic pressure gradients (Gladden, 2000; Stallknecht et al., 1998).

Changes in muscle volume may alter the force generating potential of muscle. Studies on isolated muscles have demonstrated an increase in passive muscle tension with increased muscle fluid volume (Sleboda & Roberts, 2017). Passive muscle force at a given length changes in proportion to volume change, and a measurable change in force can be observed with volume changes as small as 5% in isolated muscles (Sleboda, Wold, & Roberts, 2019). Changes in muscle volume may also influence active contractile force via influences on muscle pressure (Sleboda & Roberts, 2020). We speculate that the changes in muscle volume observed in the present study may have been accompanied by changes in the passive mechanical behavior of running muscles. Passive force development contributes considerably to moment and power generation in human locomotion (Whittington, Silder, Heiderscheit, & Thelen, 2008), and changes in passive muscle tension have the potential to influence the kinematics and energetics of locomotion. Whittington et al. (Whittington et al., 2008) demonstrated that passive force contributions occur at the end ranges of joint motions (i.e., peak hip extension, peak knee extension, and peak ankle dorsiflexion), and so fluid‐related changes in passive muscle mechanics have the greatest potential to influence force development during the late swing and early stance phase in running, potentially altering the foot strike pattern of runners (Willwacher, Regniet, Fischer, Oberländer, & Brüggemanna, 2014). Combining interventions that affect muscle fluid content in humans in vivo with measurements of passive force development seems to be an essential step in future studies. Potentially, this could address other muscle groups, for example, the hip flexors.

However, the present study faces several limitations. We did not standardize the running speed, for example, to ${\dot{V}O}_{2}\max$. Future studies should more carefully address this issue in order to develop the dose–response relationship between running exercise and muscle fluid changes. Related to this, we also did not determine total body water losses due to sweat by, for example, measuring body mass losses after the running protocol. In order to better understand the reductions in muscle ACSA at the later stages of prolonged running, future research needs to address this.

Furthermore, while there is good evidence from animal muscle preparations suggesting that changes in muscle fluid volume are related to passive stiffness (Sleboda & Roberts, 2017; Sleboda et al., 2019), we did not measure changes in passive muscle (or total joint) stiffness. Since these measurements take time, and we did not want to interrupt the running protocol longer than needed for the MRI measurements, we did not include them in our experimental protocol. However, future studies could address this relationship with dedicated experimental designs. Finally, it might be beneficial to quantify the runners' biomechanics during prolonged running. Recent research suggests that energy generation patterns within the lower extremity can change throughout a prolonged run (Sanno, Willwacher, Epro, & Brüggemann, 2018). In order to differentiate whether fluid shifts away from muscle tissue are due to dehydration or due to altered muscle activity, experimental designs should consider changes in running mechanics.

In conclusion, we provide evidence that calf muscle fluid volumes change throughout a prolonged running protocol, which might affect the force generation ability of muscle through biochemical and biomechanical pathways.

## 5 PERSPECTIVE

Calf muscle volumes undergo initial increase and subsequent decrease during prolonged, moderate intensity running in young, healthy, recreational runners. Recent evidence suggests that muscle fluid volume changes can affect passive force development and therefore the physiological performance of muscles (Sleboda & Roberts, 2017; Sleboda et al., 2019). Consequently, next to the well‐known importance of fluid shifts for cell biochemistry and cardiovascular performance (Maughan, 2010), the biomechanical effects of fluid shifts should be considered during sporting activities. This finding might have important implications for understanding distance running performance as well as injury development.

## CONFLICT OF INTEREST

None declared.

## References

[phy214414-bib-0001] Adams, G. R. , Duvoisin, M. R. , & Dudley, G. A. (1992). Magnetic resonance imaging and electromyography as indexes of muscle function. Journal of Applied Physiology, 73, 1578–1583. 10.1152/jappl.1992.73.4.1578 1447107

[phy214414-bib-0002] Alway, S. E. , MacDougall, J. D. , Sale, D. G. , Sutton, J. R. , & McComas, A. J. (1988). Functional and structural adaptations in skeletal muscle of trained athletes. Journal of Applied Physiology, 64, 1114–1120. 10.1152/jappl.1988.64.3.1114 3366734

[phy214414-bib-0003] Archer, B. T. , Fleckenstein, J. L. , Bertocci, L. A. , Haller, R. G. , Barker, B. , Parkey, R. W. , & Peshock, R. M. (1992). Effect of perfusion on exercised muscle: MR imaging evaluation. Journal of Magnetic Resonance Imaging, 2, 407–413. 10.1002/jmri.1880020409 1633393

[phy214414-bib-0004] Azizi, E. , Brainerd, E. L. , & Roberts, T. J. (2008). Variable gearing in pennate muscles. PNAS, 105, 1745–1750. 10.1073/pnas.0709212105 18230734 PMC2234215

[phy214414-bib-0005] Azizi, E. , Deslauriers, A. R. , Holt, N. C. , & Eaton, C. E. (2017). Resistance to radial expansion limits muscle strain and work. Biomechanics and Modeling in Mechanobiology, 16, 1633–1643. 10.1007/s10237-017-0909-3 28432448 PMC5889119

[phy214414-bib-0006] Björnberg, J. (1990). Forces involved in transcapillary fluid movement in exercising cat skeletal muscle. Acta Physiologica Scandinavica, 140, 221–236. 10.1111/j.1748-1716.1990.tb08994.x 2267951

[phy214414-bib-0007] Cole, W. C. , Leblanc, A. D. , & Jhingran, S. G. (1993). The origin of biexponential T2 relaxation in muscle water. Magnetic Resonance in Medicine, 29, 19–24. 10.1002/mrm.1910290106 8419738

[phy214414-bib-0008] Costill, D. L. (1977). Sweating: Its composition and effects on body fluids. Annals of the New York Academy of Sciences, 301, 160–174. 10.1111/j.1749-6632.1977.tb38195.x 270913

[phy214414-bib-0009] Costill, D. L. , Coté, R. , Fink, W. J. , & Handel, P. V. (1981). Muscle water and electrolyte distribution during prolonged exercise. International Journal of Sports Medicine, 02, 130–134. 10.1055/s-2008-1034598 7333748

[phy214414-bib-0010] Damon, B. M. , Gregory, C. D. , Hall, K. L. , Stark, H. J. , Gulani, V. , & Dawson, M. J. (2002). Intracellular acidification and volume increases explain R2 decreases in exercising muscle. Magnetic Resonance in Medicine, 47, 14–23. 10.1002/mrm.10043 11754438

[phy214414-bib-0011] Eng, C. M. , Azizi, E. , & Roberts, T. J. (2018). Structural determinants of muscle gearing during dynamic contractions. Integrative and Comparative Biology, 58, 207–218. 10.1093/icb/icy054 29889236 PMC6104701

[phy214414-bib-0012] Fisher, M. J. , Meyer, R. A. , Adams, G. R. , Foley, J. M. , & Potchen, E. J. (1990). Direct relationship between proton T2 and exercise intensity in skeletal muscle MR images. Investigative Radiology, 25, 480–485. 10.1097/00004424-199005000-00003 2345077

[phy214414-bib-0013] Fleckenstein, J. , Bertocci, L. , Nunnally, R. , Parkey, R. , & Peshock, R. (1989). 1989 ARRS Executive Council Award. Exercise‐enhanced MR imaging of variations in forearm muscle anatomy and use: importance in MR spectroscopy. American Journal of Roentgenology, 153(4), 693–698. 10.2214/ajr.153.4.693 2773724

[phy214414-bib-0014] Fleckenstein, J. L. , Haller, R. G. , Lewis, S. F. , Archer, B. T. , Barker, B. R. , Payne, J. , … Peshock, R. M. (1991). Absence of exercise‐induced MRI enhancement of skeletal muscle in McArdle’s disease. Journal of Applied Physiology, 71, 961–969. 10.1152/jappl.1991.71.3.961 1757335

[phy214414-bib-0015] Folkow, B. , Haglund, U. , Jodal, M. , & Lundgren, O. (1971). Blood flow in the calf muscle of man during heavy rhythmic exercise. Acta Physiologica Scandinavica, 81, 157–163. 10.1111/j.1748-1716.1971.tb04887.x 5552789

[phy214414-bib-0016] Gindre, J. , Takaza, M. , Moerman, K. M. , & Simms, C. K. (2013). A structural model of passive skeletal muscle shows two reinforcement processes in resisting deformation. Journal of the Mechanical Behavior of Biomedical Materials, 22, 84–94. 10.1016/j.jmbbm.2013.02.007 23587721

[phy214414-bib-0017] Gladden, L. B. (2000). Muscle as a consumer of lactate. Medicine and Science in Sports and Exercise, 32, 764–771. 10.1097/00005768-200004000-00008 10776895

[phy214414-bib-0018] Greenleaf, J. E. , Convertino, V. A. , Stremel, R. W. , Bernauer, E. M. , Adams, W. C. , Vignau, S. R. , & Brock, P. J. (1977). Plasma [Na+], [Ca2+], and volume shifts and thermoregulation during exercise in man. Journal of Applied Physiology, 43, 1026–1032. 10.1152/jappl.1977.43.6.1026 606687

[phy214414-bib-0019] Hamner, S. R. , Seth, A. , & Delp, S. L. (2010). Muscle contributions to propulsion and support during running. Journal of Biomechanics, 43, 2709–2716. 10.1016/j.jbiomech.2010.06.025 20691972 PMC2973845

[phy214414-bib-0020] Höjensgàrd, I. C. , & Stürup, H. (1953). Static and dynamic pressures in superficial and deep veins of the lower extremity in man. Acta Physiologica Scandinavica, 27, 49–67. 10.1111/j.1748-1716.1953.tb00923.x 13007498

[phy214414-bib-0021] Hudelmaier, M. , Wirth, W. , Himmer, M. , Ring‐Dimitriou, S. , Sänger, A. , & Eckstein, F. (2010). Effect of exercise intervention on thigh muscle volume and anatomical cross‐sectional areas—Quantitative assessment using MRI. Magnetic Resonance in Medicine, 64, 1713–1720. 10.1002/mrm.22550 20665894

[phy214414-bib-0022] Jenner, G. , Foley, J. M. , Cooper, T. G. , Potchen, E. J. , & Meyer, R. A. (1994). Changes in magnetic resonance images of muscle depend on exercise intensity and duration, not work. Journal of Applied Physiology, 76, 2119–2124. 10.1152/jappl.1994.76.5.2119 8063675

[phy214414-bib-0023] Jensen, B. R. , Jørgensen, K. , & Sjøgaard, G. (1994). The effect of prolonged isometric contractions on muscle fluid balance. European Journal of Applied Physiology and Occupational Physiology, 69(5), 439–444. 10.1007/BF00865409 7875142

[phy214414-bib-0024] Kjellmer, I. (1964). The effect of exercise on the vascular bed of skeletal muscle. Acta Physiologica Scandinavica, 62, 18–30. 10.1111/j.1748-1716.1964.tb03947.x 14210261

[phy214414-bib-0025] Lorenzo, I. , Serra‐Prat, M. , & Yébenes, J. C. (2019). The role of water homeostasis in muscle function and frailty: A review. Nutrients, 11, 1857 10.3390/nu11081857 PMC6723611 31405072

[phy214414-bib-0026] Lundvall, J. , Mellander, S. , Westling, H. , & White, T. (1972). Fluid transfer between blood and tissues during exercise. Acta Physiologica Scandinavica, 85, 258–269. 10.1111/j.1748-1716.1972.tb05259.x 5049421

[phy214414-bib-0027] Maughan, R. J. (2010). Distance running in hot environments: A thermal challenge to the elite runner. Scandinavian Journal of Medicine & Science in Sports, 20, 95–102. 10.1111/j.1600-0838.2010.01214.x 21029196

[phy214414-bib-0028] McColl, R. W. , Fleckenstein, J. L. , Bowers, J. , Theriault, G. , & Peshock, R. M. (1992). Three‐dimensional reconstruction of skeletal muscle from MRI. Computerized Medical Imaging and Graphics, 16, 363–371. 10.1016/0895-6111(92)90054-D 1468070

[phy214414-bib-0029] Meyer, R. A. , & Prior, B. M. (2000). Functional magnetic resonance imaging of muscle. Exercise and Sport Sciences Reviews, 28(2), 89–92. 10902092

[phy214414-bib-0030] Nicolaides, A. N. , & Zukowski, A. J. (1986). The value of dynamic venous pressure measurements. World Journal of Surgery, 10, 919–924. 10.1007/BF01658640 3798938

[phy214414-bib-0031] Nose, H. , Mack, G. W. , Shi, X. R. , & Nadel, E. R. (1988). Shift in body fluid compartments after dehydration in humans. Journal of Applied Physiology, 65, 318–324. 10.1152/jappl.1988.65.1.318 3403475

[phy214414-bib-0032] Orgiu, S. , Lafortuna, C. L. , Rastelli, F. , Cadioli, M. , Falini, A. , & Rizzo, G. (2016). Automatic muscle and fat segmentation in the thigh from T1‐Weighted MRI. Journal of Magnetic Resonance Imaging, 43, 601–610. 26268693 10.1002/jmri.25031

[phy214414-bib-0033] Ploutz‐Snyder, L. L. , Convertino, V. A. , & Dudley, G. A. (1995). Resistance exercise‐induced fluid shifts: Change in active muscle size and plasma volume. American Journal of Physiology‐Regulatory, Integrative and Comparative Physiology, 269, R536–R543. 10.1152/ajpregu.1995.269.3.R536 7573553

[phy214414-bib-0034] Ploutz‐Snyder, L. L. , Nyren, S. , Cooper, T. G. , Potchen, E. J. , & Meyer, R. A. (1997). Different effects of exercise and edema on T2 relaxation in skeletal muscle. Magnetic Resonance in Medicine, 37, 676–682. 10.1002/mrm.1910370509 9126941

[phy214414-bib-0035] Pollack, A. A. , & Wood, E. H. (1949). Venous pressure in the saphenous vein at the ankle in man during exercise and changes in posture. Journal of Applied Physiology, 1, 649–662. 18124797 10.1152/jappl.1949.1.9.649

[phy214414-bib-0036] Pons, C. , Borotikar, B. , Garetier, M. , Burdin, V. , Salem, D. B. , Lempereur, M. , & Brochard, S. (2018). Quantifying skeletal muscle volume and shape in humans using MRI: A systematic review of validity and reliability. PLoS ONE, 13, e0207847 10.1371/journal.pone.0207847 30496308 PMC6264864

[phy214414-bib-0037] Prior, B. M. , Ploutz‐Snyder, L. L. , Cooper, T. G. , & Meyer, R. A. (2001). Fiber type and metabolic dependence of T2 increases in stimulated rat muscles. Journal of Applied Physiology, 90, 615–623. 10.1152/jappl.2001.90.2.615 11160061

[phy214414-bib-0038] Raja, M. K. , Raymer, G. H. , Moran, G. R. , Marsh, G. , & Thompson, R. T. (2006). Changes in tissue water content measured with multiple‐frequency bioimpedance and metabolism measured with 31P‐MRS during progressive forearm exercise. Journal of Applied Physiology, 101, 1070–1075. 16794019 10.1152/japplphysiol.01322.2005

[phy214414-bib-0039] Rapp, G. , Ashley, C. C. , Bagni, M. A. , Griffiths, P. J. , & Cecchi, G. (1998). Volume changes of the myosin lattice resulting from repetitive stimulation of single muscle fibers. Biophysical Journal, 75, 2984–2995. 10.1016/S0006-3495(98)77739-2 9826618 PMC1299969

[phy214414-bib-0040] Sanno, M. , Willwacher, S. , Epro, G. , & Brüggemann, G. P. (2018). Positive work contribution shifts from distal to proximal joints during a prolonged run. Medicine and Science in Sports and Exercise, 50, 2507–2517. 10.1249/MSS.0000000000001707 30169401

[phy214414-bib-0041] Senay, L. C. (1970). Movement of water, protein and crystalloids between vascular and extravascular compartments in heat‐exposed men during dehydration and following limited relief of dehydration. The Journal of Physiology, 210, 617–635. 10.1113/jphysiol.1970.sp009231 5532905 PMC1395616

[phy214414-bib-0042] Senay, L. C. (1972). Changes in plasma volume and protein content during exposures of working men to various temperatures before and after acclimitization to heat: Separation of the roles of cutaneous and skeletal muscle circulation. The Journal of Physiology, 224, 61–81. 10.1113/jphysiol.1972.sp009881 5040008 PMC1331526

[phy214414-bib-0043] Senay, L. C. J. , & Pivarnik, J. M. (1985). Fluid shifts during exercise. Exercise and Sport Sciences Reviews, 13, 335. 3891371

[phy214414-bib-0044] Shellock, F. G. , Fukunaga, T. , Mink, J. H. , & Edgerton, V. R. (1991). Acute effects of exercise on MR imaging of skeletal muscle: Concentric vs eccentric actions. American Journal of Roentgenology, 156, 765–768. 10.2214/ajr.156.4.2003443 2003443

[phy214414-bib-0045] Shi, J. , Zheng, Y. P. , Chen, X. , & Huang, Q. H. (2007). Assessment of muscle fatigue using sonomyography: Muscle thickness change detected from ultrasound images. Medical Engineering & Physics, 29, 472–479. 10.1016/j.medengphy.2006.07.004 16908212

[phy214414-bib-0046] Siebert, T. , Günther, M. , & Blickhan, R. (2012). A 3D‐geometric model for the deformation of a transversally loaded muscle. Journal of Theoretical Biology, 298, 116–121. 10.1016/j.jtbi.2012.01.009 22251888

[phy214414-bib-0047] Sjogaard, G. , & Saltin, B. (1982). Extra‐ and intracellular water spaces in muscles of man at rest and with dynamic exercise. American Journal of Physiology‐Regulatory, Integrative and Comparative Physiology, 243, R271–R280. 10.1152/ajpregu.1982.243.3.R271 7114288

[phy214414-bib-0048] Sleboda, D. A. , & Roberts, T. J. (2017). Incompressible fluid plays a mechanical role in the development of passive muscle tension. Biology Letters, 13, 20160630 10.1098/rsbl.2016.0630 28123108 PMC5310577

[phy214414-bib-0049] Sleboda, D. A. , & Roberts, T. J. (2020). Internal fluid pressure influences muscle contractile force. Proceedings of the National Academy of Sciences, 117(3), 1772–1778. 10.1073/pnas.1914433117 PMC6983394 31879350

[phy214414-bib-0050] Sleboda, D. A. , Wold, E. S. , & Roberts, T. J. (2019). Passive muscle tension increases in proportion to intramuscular fluid volume. Journal of Experimental Biology, 222(21), jeb209668 10.1242/jeb.209668 31558592 PMC6857584

[phy214414-bib-0051] Stallknecht, B. , Vissing, J. , & Galbo, H. (1998). Lactate production and clearance in exercise. Effects of training. A mini‐review. Scandinavian Journal of Medicine & Science in Sports, 8, 127–131. 10.1111/j.1600-0838.1998.tb00181.x 9659671

[phy214414-bib-0052] van Beaumont, W. , Underkofler, S. , & van Beaumont, S. (1981). Erythrocyte volume, plasma volume, and acid‐base changes in exercise and heat dehydration. Journal of Applied Physiology, 50, 1255–1262. 10.1152/jappl.1981.50.6.1255 7263386

[phy214414-bib-0053] von Duvillard, S. P. , Braun, W. A. , Markofski, M. , Beneke, R. , & Leithäuser, R. (2004). Fluids and hydration in prolonged endurance performance. Nutrition, 20, 651–656. 10.1016/j.nut.2004.04.011 15212747

[phy214414-bib-0054] Ward, D. S. , Hamilton, M. T. , & Watson, P. D. (1996). Measurement of tissue volume during non‐steady state high‐intensity muscle contraction. American Journal of Physiology‐Regulatory, Integrative and Comparative Physiology, 271, R1682–R1690. 10.1152/ajpregu.1996.271.6.R1682 8997370

[phy214414-bib-0055] Warfield, S. K. , Mulkern, R. V. , Winalski, C. S. , Jolesz, F. A. , & Kikinis, R. (2000). An image processing strategy for the quantification and visualization of exercise‐induced muscle MRI signal enhancement. Journal of Magnetic Resonance Imaging, 11, 525–531. 10.1002/(SICI)1522-2586(200005)11:5<525:AID-JMRI8>3.0.CO;2-2 10813862

[phy214414-bib-0056] Watson, P. D. , Garner, R. P. , & Ward, D. S. (1993). Water uptake in stimulated cat skeletal muscle. American Journal of Physiology‐Regulatory, Integrative and Comparative Physiology, 264, R790–R796. 10.1152/ajpregu.1993.264.4.R790 8476122

[phy214414-bib-0057] Whittington, B. , Silder, A. , Heiderscheit, B. , & Thelen, D. G. (2008). The contribution of passive‐elastic mechanisms to lower extremity joint kinetics during human walking. Gait & Posture, 27, 628–634. 10.1016/j.gaitpost.2007.08.005 17928228 PMC2505349

[phy214414-bib-0058] Williams, C. , & Nute, M. L. (1983). Some physiological demands of a half‐marathon race on recreational runners. British Journal of Sports Medicine, 17, 152–161. 10.1136/bjsm.17.3.152 PMC1859178 6652396

[phy214414-bib-0059] Willwacher, S. , Regniet, L. , Fischer, K. M. , Oberländer, K. D. , & Brüggemanna, G. P. (2014). The effect of shoes, surface conditions and sex on leg geometry at touchdown in habitually shod runners. Footwear Science, 6, 129–138. 10.1080/19424280.2014.896952

